# Differential protein profiling of soil diazotroph *Rhodococcus qingshengii* S10107 towards low-temperature and nitrogen deficiency

**DOI:** 10.1038/s41598-019-56592-8

**Published:** 2019-12-30

**Authors:** Deep Chandra Suyal, Divya Joshi, Saurabh Kumar, Ravindra Soni, Reeta Goel

**Affiliations:** 1 Department of Microbiology, Akal College of Basic Sciences, Eternal University, Baru Sahib, Sirmaur, 173101 Himachal Pradesh India; 2 Department of Microbiology, College of Basic Sciences and Humanities, G.B.Pant University of Agriculture and Technology, Pantnagar, 263145 Uttarakhand India; 3 Department of Agricultural Microbiology, College of Agriculture, Indira Gandhi Krishi Viswavidyalaya, Raipur, C.G. India

**Keywords:** Bacteria, Applied microbiology

## Abstract

Protein-based biomarkers can be a promising approach for identification and real-time monitoring of the bio-inoculants employed under sustainable agricultural plans. In this perspective, differential proteomics of psychrophilic diazotroph *Rhodococcus qingshengii* S10107 (JX173283) was performed to unravel its adaptive responses towards low-temperature nitrogen deficiency and identification of a biomarker for respective physiological conditions. LC-MS/MS-based proteome analysis mapped more than 4830 proteins including 77 up-regulated and 47 down-regulated proteins (p ≤ 0.05). Differential expression of the structural genes of *nif* regulon viz. *nif*H, *nif*D, and *nif*K along with their response regulators i.e. *nif*A, *nif*L, and *nif*B indicated that the nitrogenase complex was activated successfully. Besides up-regulating the biosynthesis of certain amino acids *viz*. Leucine, Lysine, and Alanine; the expression of the peptidoglycan synthesis proteins were also increased; while, the enzymes involved in Lipid biosynthesis were found to decrease. Furthermore, two important enzymes of the pentose phosphate pathway *viz*. *Transketolase* and *Transaldolase* along with *Ribose import ATP-binding protein RbsA* were also found to induce significantly under low temperature a nitrogen deficient condition, which suggests the cellular need for ample ribose sugar instantly. Additionally, comparative protein profiling of S10107 strain with our previous studies revealed that *CowN* protein was significantly up-regulated in all the cases under low-temperature nitrogen deficient conditions and therefore, can be developed as a biomarker. Conclusively, present study for the first time provides an in-depth proteome profiling of *R. qingshengii* S10107 and proclaims *Cow*N as a potential protein biomarker for monitoring BNF under cold niches.

## Introduction

Nitrogen is an essential nutrient for crop growth and development. In spite of having a largest atmospheric reservoir, its soil availability is very low and thus, mostly requires external inputs. Biofertilizers are among the effective and eco-friendly alternative available in the respective sector. Global Industry Report revealed that their market size was USD 787.8 million in 2016 including diazotrophs as the largest segment with 75.0% of global revenue share^1^.

Recent trends indicating that microbial biofertilizers are unable to confer expected benefits to the farmers. However, they should not be blamed at first glance, because, it might be the result of their adaptive failure towards natural field conditions as well as local edaphic factors. Instead, efforts should be made to develop such technologies which can monitor the real-time performance of the bioinoculant(s) so that they can be replaced on time and loss of the farmer’s can be minimized. Under *in situ* conditions, biological nitrogen fixation (BNF) process can be monitored by using acetylene reduction assay (ARA)^2^, ^15^N_2_ assimilation technique and gene probes^2,3^. Besides having their own limitations, these techniques require sophisticated instrumentations and skilled personals which restrict their implementation. An alternative can be the microbial proteins for their identification and tracking functional interactions within a niche^4,5^. Such protein-based biomarker technologies are already being commercialized for clinical purpose^6^, bioremediation^7^ and heavy metal detection^8^; but, in case of agriculture, they are still in infancy.

A series of proteomic investigations have been performed by the author group on different diazotrophs *viz*. *Dyadobacter psychrophilus* B2 and *Pseudomonas jessenii* MP1^9^; *P. palleroniana* N26-GL^10^; *P. palleroniana* N26-GB^11^ and *P. migulae* S10724^12^ under low temperature nitrogen deficient conditions to identify the associated protein biomarker(s). In this series, for the first time, the proteome of psychrophilic and diazotrophic actinobacterium *R. qingshengii* S10107 is being analyzed. Besides exploring the adaptive mechanisms of diazotrophic actinomycete towards cold diazotrophy, present study provides a concluding remark to the previous outcomes and proclaims *Cow*N as a potential protein biomarker for monitoring BNF under cold niches. Further, on the contrary to other cold adapted diazotrophs, *R. qingshengii* S10107 has displayed wide ranges of metabolic responses which could be the active area of investigation in future. Being a potential plant growth promoting strain^13^, *R. qingshengii* S10107 can also be explored for hill agricultural sustainability and zero budget farming.

## Results

The proteome of *R. qingshengii* S10107 was analyzed under two different physiological conditions i.e. low-temperature nitrogen deficient condition (without nitrogen source; BM) and low-temperature nitrogen sufficient condition (with nitrogen source, NB). The analysis was performed with three biological and three technical replicates.

### Identification of the differentially expressed proteins

**Figure 1:**
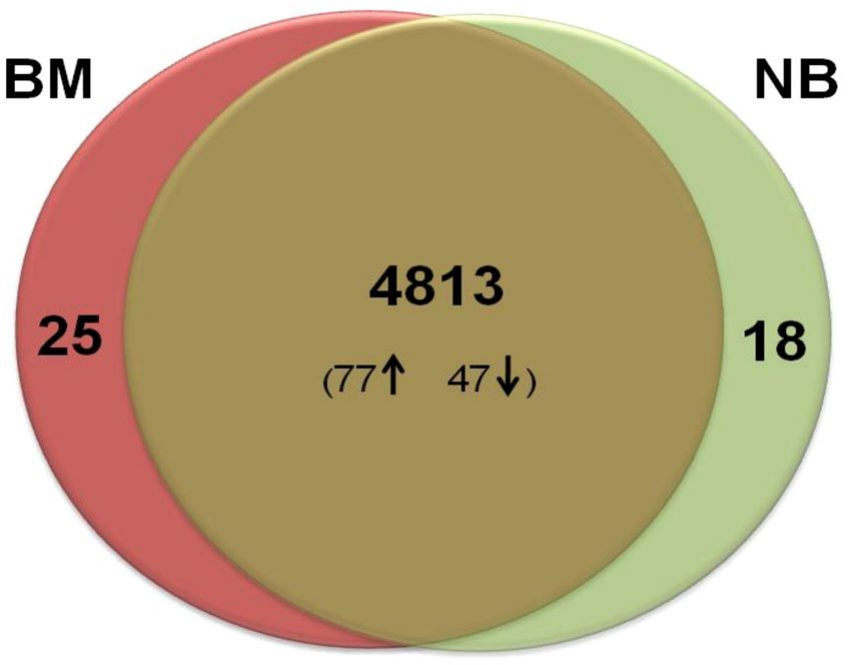
Venn diagram depicting the total number of expressed proteins of *Rhodococcus qingshengii* S10107 strain under Nitrogen deficient (BM) and Nitrogen sufficient (NB) conditions. Cells were harvested at mid-logarithmic phase. A total of 4813 proteins are shared by both BM and NB, whereas the BM (25) showed more exclusive proteins than the NB (18). The analysis was performed in triplicates. The diagram shows the average value of proteins obtained from three replicates of the experiment.

The whole proteomic dataset mapped to a total of 4838 proteins under BM and 4831 proteins under NB with a 1% false detection rate (FDR) (Fig. 1). There were 4813 proteins shared between the BM and NB along with 25 exclusive proteins under BM, and 18 under NB (p ≤ 0.05). Moreover, 77 proteins were found significantly up-regulated, while, 47 were down-regulated with ≥2-fold change under BM in reference to its counterpart.

### Protein-protein interaction (PPI) network analysis

**Figure 2:**
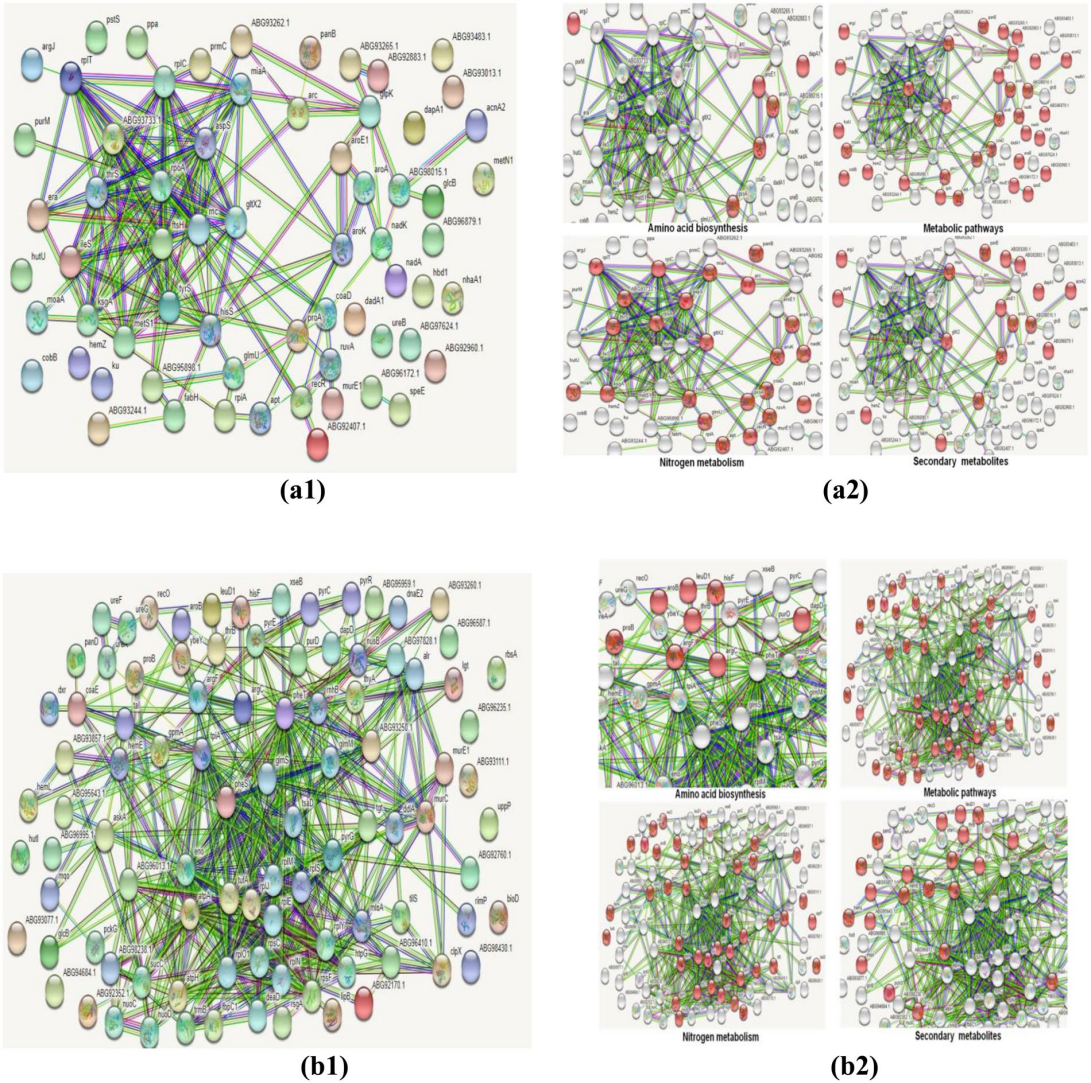
Protein-Protein Interaction (PPI) networks of differentially expressed proteins (with 2-fold or greater change) in NB (**a**) and BM (**b**). The networks a1 & b1 represent the total proteins expressed while a2 & b2 highlight the proteins involves in various processes. The protein-protein interactions were analyzed using the String database (version 9.1, Available online: http://string-db.org/) with the confidence score > 0.7. The analysis was performed in triplicates on the average value of proteins obtained from three replicates of the experiment.

Protein-protein interactions (PPIs) are considered very critical for cell survival. They are essential for the understanding of cellular physiology under different conditions. Therefore, PPI network analysis was performed to emphasize the crucial proteins involved in low-temperature nitrogen fixation (Fig. 2). Among BM proteins, *ArgF (Ornithine carbamoyltransferase, Q8YMM6); HisF (Imidazole glycerol phosphate synthase subunit, A8HYT7)*; *ProB (Glutamate 5-kinase, B2JHD6); Eno (Enolase, B2JIX0); PyrG (CTP synthase, B7KF08); PheT (Phenylalanine— tRNA ligase beta subunit, Q8YMH5); PheS (Phenylalanine—tRNA ligase alpha subunit, A0A1D8TAJ1); GlmS (Glutamine—fructose-6-phosphate aminotransferase, P59362)* and *EcaA (Carbonic anhydrase, P94170)* were identified as hub nodes along with higher BC values (Table 1). Besides them, *AtpA (ATP synthase subunit alpha, Q98EV6); AtpH (ATP synthase subunit delta, A8HS18); UppP (Undecaprenyl-diphosphatase, Q89WH1); DeaD (ATP-dependent RNA helicase, V9XLR7); TpiA (Triosephosphate isomerase, Q8YP17); TufA (Elongation factor Tu, C0ZVT7); PckG (Phosphoenolpyruvate carboxykinase, B2JJT8); Tkt (Transketolase, Q8YRU9) and SucC (Succinate-CoA ligase, A1KAU3)* were identified as hub nodes only while, *LeuD1 (3-isopropyl malate dehydratase, Q98E51); DnaE (Error-prone DNA polymerase, Q98E34); DdlA (D-alanine-D-alanine ligase B2JHF8); Tal (Transaldolase, P58561); UreF (Urease accessory protein, B5XU25); UreG (Urease accessory protein, A1KBB1) and UreA (Urease subunit gamma, Q8YQZ3)* had large BC values. However, nine proteins *viz*. *NifH (Nitrogenase iron protein, C7SI80), NifA (nif-specific regulatory protein, P56266), NifL (Nitrogen fixation regulatory protein, P06772), NifB (FeMo cofactor biosynthesis protein, P09825), NifD (Nitrogenase molybdenum-iron protein, P06120), NifK (Nitrogenase molybdenum-iron protein, P07329), NirS (Nitrite reductase, P24474), CowN (N*_*2*_*-fixation sustaining protein, C1DIY8)* and *CooA (Carbon monoxide oxidation transcription regulator, C1DIY7)* did not appear in a match with *R. qingshengii* proteins, thereby, indicating towards the unavailability of respective proteins in the database (Supplementary material).

**Table 1:** Significant key nodes of the PPI Network*.

Parameters	Gene Names	
	Nitrogen Stress (BM)	Nitrogen Sufficient (NB)
(Hub + Large BC) nodes	*arg*F*, his*F, *eca*A, *eno, pyr*G, *phe*T, *phe*S, *glm*S, *pro*B	*pro*A, *aro*A, *aro*K, *fts*H, *thr*S, *rpo*A, *ksg*A, *asp*S, *his*S, *mia*A
Hub nodes	*arg*F*, eno, his*F, *pyr*G, *phe*T, *phe*S, *glm*S, *upp*P, *eca*A, *dea*D, *tpi*A*, tuf*A*, atp*A, *atp*H, *tkt, pck*G, *suc*C, *pro*B	*ile*S, *rpl*C, *glc*B, *glt*X2, *rnc*, *met*S1, *rplT, pro*A, *aro*A, *aro*K, *fts*H, *thr*S, *rpo*A, *ksg*A, *asp*S, *his*S, *mia*A
Large BC Nodes	*arg*F*, his*F, *leu*D_1_, *dna*E, *glm*S, *ddl*A, *tal, ure*F, *ure*G, *ure*A, *eno, pyr*G, *phe*T, *phe*S, *pro*B, *eca*A	*cob*B, *mur*E1, *hbd*A, *ure*B, *hem*Z, ABG93733.1 *(fus*A*), rpo*A, *pro*A, *aro*A, *aro*K, *fts*H, *thr*S, *rpo*A, *ksg*A, *asp*S, *his*S, *mia*A

*As revealed by PPI network analysis (SM).

In case of NB, *ProA (Gamma-glutamyl phosphate reductase, Q89X85); AroA (3-phosphoshikimate 1-carboxyvinyltransferase, C1B1F1); AroK (Shikimate kinase Q98FY0); FtsH (ATP-dependent zinc metalloprotease, A0A059MKS5); ThrS (Threonine- tRNA ligase, Q0S1E2); RpoA (DNA-directed RNA polymerase subunit alpha, Q0S3E7); KsgA (rRNA small subunit methyltransferase A, A0A379MN38); AspS (Aspartate- tRNA ligase, Q0S0P3); HisS (Histidine- tRNA ligase; Q0S1B6) and MiaA (tRNA dimethylallyltransferase, B2JGD1)* were recognized as hub as well as higher BC nodes. Proteins *IleS (Isoleucine— tRNA ligase, U0FHY2); RplC (50S ribosomal protein L3, X0QHH3); GlcB (Acyl-[acyl-carrier-protein]—UDP-N-acetylglucosamine O-acyltransferase, A8I491); GltX2 (Glutamate- tRNA ligase, A0A1F2PR92); Rnc (Ribonuclease 3, Q0S2E1); MetS1 (Methionine— tRNA ligase, Q0S4U4)*, and *RplT (50S ribosomal protein L20, Q0SI47)* were only hubs; while*, FusA (Elongation factor G, Q0SFF3); CobB (Hydrogenobyrinate a,c-diamide synthase, Q98KP1); MurE1 (UDP-N-acetylmuramoyl-L-alanyl-D-glutamate—2,6-diaminopimelate ligase, Q89FU2); HbdA (3-hydroxybutyryl-CoA dehydrogenase, Q45223); ureB (Urease subunit beta, Q98CY6); hemZ (Ferrochelatase, Q0S0F7) and rpoA (DNA-directed RNA polymerase subunit alpha, Q0S3E7) were found to have higher BC under NB*.

### GO-based functional characterization of the differentially expressed proteins

**Figure 3:**
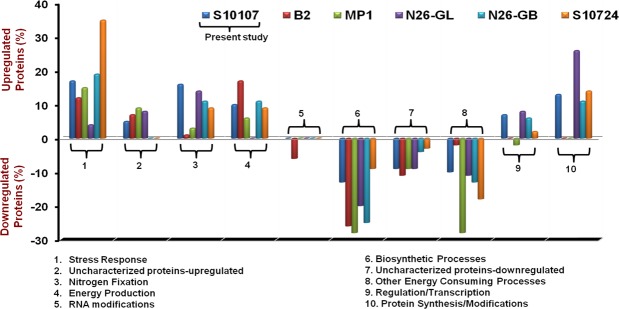
Comparative protein profiling among *Rhodococcus qingshengii* S10107 (present study); *Dyadobacter psychrophilus* B2 and *P. jessenii* MP1^9^; *P. palleroniana* N26-GL^10^; *P. palleroniana* N26-GB^11^ and *P. migulae* S10724^12^ under low temperature N_2_ fixing conditions, according to their biological functions.

All the differentially expressed proteins were characterized on the basis of their biological functions and compared with the earlier studies (Fig. 3). Proteins associated with stress response, nitrogen fixation and energy production were up-regulated while that of biosynthetic processes and energy consuming processes were observed to down-regulate by all the diazotrophs studied under low-temperature nitrogen deficient conditions. Besides them, a good fraction of the up-regulated and down-regulated proteins were found uncharacterized which need a detailed investigation in the future. In case of the present study, 17% of the proteins were related to stress response, followed by nitrogen fixation (16%), protein synthesis/modifications (13%), energy production (10%), gene regulation/transcription (7%) and uncharacterized (5%). Among down-regulated proteins, the majority of the proteins were involved in biosynthetic processes (13%) and energy consuming processes (10%) besides uncharacterized proteins (9%).

Additionally, functional enrichments in the PPI network were explored to provide a more specific description of the differentially expressed proteins in terms of their involvement in amino acid biosynthesis (AAB); metabolic pathways (MP); nitrogen metabolism (NM) and production of secondary metabolites (Fig. 2).

#### Amino acid biosynthesis (AAB)

Proteins *LeuD1, ArgF, ArgC (N-acetyl-gamma-glutamyl-phosphate reductase, B7JY20), HisF, Putative glutamate—cysteine ligase (AZC2303,A8I5N7), ProB, ThrB (Homoserine kinase, A9HS91), DapD (2,3,4,5-tetrahydropyridine-2,6-dicarboxylate N-succinyltransferase,B2JID7), Alr (Alanine racemase,W8HAH8)* and *AroB (3-dehydroquinate synthase, Q0S0N0)* were involved in biosynthesis of different amino acids under BM, while*, DapA1 (4-hydroxy-tetrahydrodipicolinate synthase, A0A1H4N6J7), TyrS (Tyrosine— tRNA ligase, Q98NS5), IleS, ProA, AroE1 (Shikimate dehydrogenase, Q98DY3) AroA, ThrS, HisS, AspS*, and *AroK* were responsible for AAB under NB.

#### Metabolic pathways (MP)

Proteins involved in MP were *Bphy_3511 (Acetaldehyde dehydrogenase1, B2JLM7), LeuD1, UppP, PyrG, ArgF, CoaE (Dephospho-CoA kinase, Q98DY2), Lgt (Prolipoprotein diacylglyceryl transferase, A9HBY2), HisF, BioD (ATP-dependent dethiobiotin synthetase, B7K5E6), MurC (UDP-N-acetylmuramate—L-alanine ligase, B2JHF9), MloA (Protein MloA, Q8RN11), LipB (Octanoyltransferase, Q89JM6), YbeY (Endoribonuclease YbeY, Q98BK1), ProB, atpA, AtpH, ThrB, TufA, GpmA (2,3-bisphosphoglycerate-dependent phosphoglycerate mutase, B2JC95), AskA (Acetate kinase, B8HVB6), ThyA (Thymidylate synthase, Q0SEI1), RpsF (30S ribosomal protein S6), Tkt, AccC (Biotin carboxylase, Q06862), PanD (Aspartate 1-decarboxylase, Q8YR79), HutI (Imidazolonepropionase, B2JCJ1), PckG, SucC, Eno*, Urease accessory *proteins, glmM (Phosphoglucosamine mutase, Q98F91), GlmS, DdlA, Dxr (1-deoxy-D-xylulose 5-phosphate reductoisomerase, B7K5G6), Mqo (Probable malate:uinine oxidoreductase, Q89XM4), AroB, TpiA, Tal* etc. under BM and *DapA1, GlcB, NadK (NAD kinase, Q0SI70), ArgJ (Arginine biosynthesis protein, Q8YPF9), PanB (3-methyl-2-oxobutanoate hydroxymethyltransferase, Q0SHJ0), ProA, AroE1, AcpS (Holo-[acyl-carrier-protein] synthase, Q0SGU2), HbdA, RpiA (Ribose-5-phosphate isomerase A, B7K6D3), SpeE (Polyamine aminopropyltransferase), HutU (Urocanate hydratase, Q89GV4), FabH (3-oxoacyl-[acyl-carrier-protein] synthase, Q982Z8), GlmU (Bifunctional protein GlmU, Q0S4N3), Rha1_ro06238 (Isocitrate dehydrogenase, Q0S371), GlpK (Glycerol kinase, A1K962), AroA, MoaA (GTP 3*′*,8-cyclase, A0A143Q879), CobB, AroK, HemZ* etc. under NB.

#### Nitrogen metabolism (NM)

In case of Nitrogen metabolism (NM), *R. qingshengii* S10107 has up-regulated (BM) *UppP, ArgF, Lgt, HisF, BioD, MurC, MloA, LipB, YbeY, AZC2303, ProB, AtpA, AtpH, TufA, HemL (Glutamate-1-semialdehyde 2,1-aminomutase, B8HYK1), GpmA, ThyA, PanD, Eno, Ribosomal proteins and* Urease accessory proteins while, Arc *(Proteasome-associated ATPase, Q0SIF4), NadK, PanB, ProA, FusA, KsgA, GlmU, AroA, MoaA, GltX2, CoaD (Phosphopantetheine adenylyltransferase, B5XTG9), ThrS, Apt (Adenine phosphoribosyltransferase, Q0S1C1), HisS, AroK, AspS* etc. were down-regulated (NB).

#### Secondary metabolites (SM)

The proteins involved in secondary metabolite formation (SM) under BM were *LeuD1, ArgF, ArgC, MloA, YbeY, ThrB, HemL, GpmA, PanD, PurD, PckG, SucC, Eno, Dxr, Mqo, HemE, AroB, TpiA and Tal while, DapA1, argJ, Rha1_ro01056 (Probable glycogen debranching enzyme, Q0SHV3), PanB, ProA, AroE1, Rha1_ro01447 (Glycogen phosphorylase, Q0SGS1), RpiA, PurM (Phosphoribosylformylglycinamidine cyclo-ligase, Q0S760), Rha1_ro05098 (3-demethylubiquinone-9 3-O-methyltransferase, Q0S6F7), Rha1_ro06238, AroA, GltX2, MiaA, AroK, AcnA2 (Aconitate hydratase, Q0S0G5)* and *HemZ* were under NB.

### CowN as a protein biomarker

In order to identify the protein biomarker(s) associated with the nitrogen fixation process at lower temperatures, the author group has performed a series of experiments on six different cold-adapted microorganisms viz. *P. palleroniana* N26-GL^10^; *Dyadobacter psychrophilus* B2 and *P. jessenii* MP1^9^; *P. palleroniana* N26-GB^11^ and *P. migulae* S10724^12^ including present study (*R. qingshengii* S10107). Moreover, two distinct proteomic approaches i.e. gel-based^9,11,12^ and gel-less approach^10^ were also employed to rectify the experimental biases.

**Figure 4:**
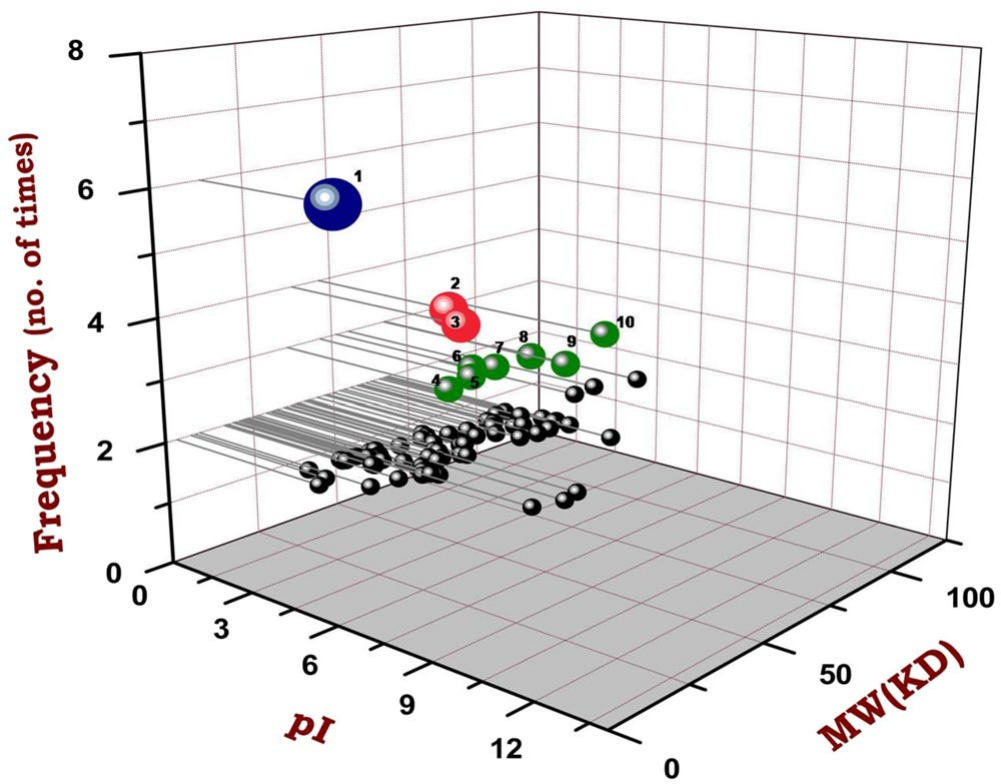
3D Scatter plot illustrating the expressional pattern of the proteins associated with low-temperature N_2_ fixation. OriginPro software was used to perform the analysis based on the Isoelectric point of the proteins (pI), molecular weight (KD) and their frequency to occur as observed during previous studies. Proteins with ≥ 3 time of occurrence are numbered *viz*. 1, *CowN*; 2, *Eno*; 3, *PyrG*; 4, *IlvC*; 5, *ClpX*; 6, *MnmE*; 7, *AtpA*; 8, *HtpG*; 9, *MutL*; 10, *IleS*.

In this perspective, comparative protein profiling of all the diazotrophs revealed that *CowN* was significantly up-regulated in all the six experimentation followed by *Eno* which was up-regulated in four cases only (Fig. 4). *PyrG* was also found to express in four instances, however, in three cases it was a down-regulated and up-regulated only once (SM). Similarly, proteins *IlvC, ClpX, MnmE, AtpA, HtpG, MutL*, and *IleS* were expressed in three cases only. Among them, AtpA, HtpG, and MutL were up-regulated in all the three cases while; *MnmE* and *IleS* were down-regulated, subsequently.

## Discussion

Genus *Rhodococcus* is well known for its broad metabolic versatility, however, the diazotrophic potential of this actinobacterium is least studied. All the previous proteomic investigations, which were performed on *Rhodococcus*, only highlighted its catabolic behavior towards different organic compounds *viz*. Fluoranthene^14^, toluene and phenol^15^, propane^16^, triacylglycerol^17^, 4,4′-Dithiodibutyric Acid^18^, benzoate^19^ and polyaromatic compounds^20^. In this perspective, the present study for the first time unravels its proteomic response towards the abiotic stress and identified a protein biomarker for low-temperature nitrogen deficient conditions.

The study revealed that *R. qingshengii* S10107 expressed all the three structural *nif* genes *viz*. *nif*D, *nif*H and *nif*K under BM along with their regulators i.e. *nif*A, *nif*L, and *nif*B; thereby, confirming its nitrogen fixation potential^21^. However, complete *nif* regulon could not be observed, which might be due to the fact that diazotrophs do not express all the *nif* genes at a time in order to minimize their energy consumption^22^. Additionally, the accumulated *Nif* proteins may get cleaved by the proteases *i.e. ClpX* (N1M9I2) as also evident from earlier studies^10^. Therefore, the kinetics of *nif* regulon must be studied over time to scan the complete network. Besides the *Nif* proteins, two associated proteins were also observed *viz. CO Weal-Nitrogenase (CowN)* and *CO-responsive regulator (CooA). CowN* is known to protect the nitrogenase from Carbon monoxide (CO) stress which is supposed to induce under low-temperature nitrogen deficient conditions^10,23,24^.

*R. qingshengii* S10107 was found to employ unique strategies for performing important cellular processes under low-temperature nitrogen deficient condition. Under BM, proteins *3-isopropylmalate dehydratase, 2,3,4,5-tetrahydropyridine-2,6-dicarboxylate N-succinyltransferase* and *Alanine racemase* were up-regulated which are responsible for the biosynthesis of the amino acids Leucine, Lysine, and Alanine, respectively. Lysine and Alanine in a peptide help it to get α-helical conformation^25^ and thus, contributed towards low-temperature adaptation^26^. Similarly, *Tyrosine-tRNA ligase, Isoleucine*–*tRNA ligase* and *Aspartate*–*tRNA(Asp/Asn) ligase* were down-regulated which are responsible for the biosynthesis of Tyrosine, Iso-leucine, and Aspartate. These amino acids tend to be decreased when the organism exposed to the cold^27^.

Further, among the up-regulated proteins, *Ornithine carbamoyltransferase (ArgF)* and *N-acetyl-gamma-glutamyl-phosphate reductase (ArgC)* are known for the biosynthesis of Arginine, which mostly serves as a key source of carbon and nitrogen for ornithine, proline, and pyrimidine^28,29^. Up-regulation of *ArgC* during low-temperature nitrogen fixation needs to be investigated in detail because of the two main reasons - first - it catalyzes high energy consuming reaction which is otherwise very rare event during such conditions^10,30^ and second – it causes growth delay as well as inefficient nodule formation in diazotrophs^30^. In the case of Proline biosynthesis, the genes encode for protein *Gamma-glutamyl phosphate reductase (ProA)* and *Glutamate 5-kinase (ProB)* are organized in a single operon i.e. *pro*BA. In the present study, S10107 strain increases the expression of *ProB* while, decreases *ProA*, thereby, regulated the intracellular concentration of Proline. *P. putida, K. aerogenes*, and *E. coli*, are known to enhance the catabolism of Proline under nitrogen-deficient conditions^29,31^. A similar condition was also observed in case of *Histidine* which showed up-regulation of *Imidazole glycerol phosphate synthase subunit* (*HisF*) and down-regulation of *Histidine*–*tRNA ligase* (*HisS*). These expressional patterns can be supported with the fact that nitrogen fixation is an energy consuming process and therefore, down-regulation and/or shutting down the nonessential proteins is pre-requisite to the cell survival.

Cold along with nitrogen deficiency represents dual-stress condition for the microorganisms involving environmental as well as nutritional stress. Therefore, S10107 strain up-regulated stress associated proteins *viz. UvrABC system protein A (Q0SI39); DNA repair protein RecO (Q985A3), Chaperone protein HtpG (Q0S467)*, and *Error-prone DNA polymerase (Q98E34). UvrABC* and *RecO* are the multienzyme systems which aid in DNA repair; while *Chaperone Htp*G protects the cellular proteins from the stress conditions^9,10^. Also, up-regulation of *Error-prone DNA polymerase, UvrABC*, and *RecO* revealed the cellular need for DNA maintenance under given physiological conditions^32,33^. Conclusively, these proteins are responsible for tight regulation of the housekeeping genes along with their protection to sustain the life under multi-stress conditions.

Under BM conditions, S10107 strain showed a distinct metabolic behavior by up-regulating the proteins of peptidoglycan synthesis (*Undecaprenyl-diphosphatase, Q89WH1* and *D-alanine*–*D-alanine ligase, B2JHF8*); Glutamine synthesis (*CTP synthase, B7KF08; Glutamine*–*fructose-6-phosphate aminotransferase, P59362*); Biotin metabolism (*ATP-dependent dethiobiotin synthetase, B7K5E6* and *Biotin carboxylase, Q06862*), Nitrogen metabolism (Urease enzyme complex) and Nucleotide metabolism (*dITP/XTP pyrophosphatase, Q98DN4 and Thymidylate synthase, Q0SEI1*) while down-regulating the enzymes for Lipid biosynthesis (*Acyl-[acyl-carrier-protein]*–*UDP-N-acetylglucosamine O-acyltransferase, A8I491* and *3-hydroxybutyryl-CoA dehydrogenase, Q45223*) and Pantothenate biosynthesis (*3-methyl-2-oxobutanoate hydroxymethyltransferase, Q0SHJ0*). Up-regulation of the peptidoglycan synthesis proteins while down-regulation of Lipid biosynthesis-related proteins revealed that *R. qingshengii* S10107 tend to thicken their cell wall to respond towards external stress. Further, enhancement of Glutamine synthesis proteins can be justified by the general tendency of the microbial cell to prefer ammonium and glutamine as a nitrogen source^29^. Furthermore, *CTP synthase* is responsible to regulate cytidine triphosphate (CTP) concentration within a cell. Killer *et al*., (2018) has developed a *CTP synthase* based tool to identify the family Bifidobacteriaceae. *Glutamine*–*fructose-6-phosphate aminotransferase* has glutaminase activity that links hexosamine biosynthetic pathway to the hexose pathway and thereby, plays a regulatory role for the nutrient-sensing system^34^.

The up-regulation of the two enzymes of the Pentose phosphate pathway *viz*. *Transketolase (Q8YRU9)* and *Transaldolase (P58561)* along with *Ribose import ATP-binding protein RbsA (Q0S9A4)* under BM suggests the cellular need of excessive ribose sugar instantly. It can again be supported with the down-regulation of *Ribose-5-phosphate isomerase (B7K6D3)* which is responsible for the conversion of ribose-5-phosphate to ribulose-5-phosphate. In addition to these, three enzymes of Gluconeogenesis *(2,3-bisphosphoglycerate-dependent phosphoglycerate mutase, B2JC95; Phosphoenolpyruvate carboxykinase, B2JJT8*, and *Triosephosphate isomerase, Q8YP17)* and two of glucose catabolism *(Enolase, B2JIX0 and Succinate*–*CoA ligase, A1KAU3)* were also found to up-regulate under BM. Contrary to these, proteins related to the glycogen metabolism *(glycogen debranching enzyme, Q0SHV3 and Glycogen phosphorylase, Q0SGS1)* and glycerol metabolism *(Glycerol kinase, A1K962)* were found down-regulated along with *Isocitrate dehydrogenase (Q0S371)*, an important enzyme of TCA cycle. It might be the part of the adaptation strategies of the microorganism so that it could utilize carbohydrates as well as non-carbohydrates precursors for fulfilling its energy demands. The similar results were also observed by Ma *et al*.^35^. This group have used SILAC method for analysis the proteome of *Edwardsiella tarda* ATCC 15947 under prolonged cold stress and reported that the enzymes of Gluconeogeneis were significantly enhanced under cold. Furthermore, Tullio *et al*.^36^ have analysed the proteome of the diazotroph *Rhizobium freirei* and observed the up-regulation of Gluconeogeneis associated enzymes under metabolic stress conditions.

The expression of CowN was reported in the proteome of all the six previously studied cold adapted diazotrophs *viz*. *P. palleroniana* N26-GL^10^; *Dyadobacter psychrophilus* B2 and *P. jessenii* MP1^9^; *P. palleroniana* N26-GB^11^ and *P. migulae* S10724^12^ including this study (*R. qingshengii* S10107) under cold and nitrogen deficient conditions. Therefore, this protein is being identified as a protein biomarker for monitoring BNF under cold niches. Several protein-based biomarkers are already being used for the clinical purpose^6^, veterinary science^37^, bioremediation^7^ and heavy metal detection^8^. Saito *et al*.^38^ detected the multiple nutrient stresses at Pacific Ocean biomes by using the protein biomarkers. Recently, Andrade-Herrera *et al*.^39^ have developed an earthworm based biomarker for pesticides and toxicity assessment in agricultural soils. However, information is not available about such biomarkers which can monitor any biogeochemical processes under abiotic stress that is going to be imperative for precision farming in the future.

In conclusion, the present study provides a detailed investigation on the adaptive responses of *R. qingshengii* S10107 towards cold diazotrophy which can be explored for further advance research. Further, *CowN* can be proclaimed as a protein biomarker for diazotrophic identification and monitoring of BNF under cold and nitrogen deficient conditions. Moreover, the role of carbon monoxide and ribose sugar in low-temperature diazotrophy should be explored for detailed investigation.

## Methods

### Bacterial strain and growth conditions

*R. qingshengii* S10107 (JX173283) was originally isolated from the rhizospheric soil of *Phaseolus vulgaris* L. (Red Kidney Bean, RKB) from Chhiplakot region (3,290 m, 30.06 °N, 79.01 °E) of Kumaun Himalayas on the Burk’s Medium (nitrogen deficient medium) at 10 °C. This strain has already been characterized as diazotroph^12^.

### Proteome extraction and LC-MS/MS analysis

The proteome of S10107 strain was extracted at the mid-log phase in triplicates as per the earlier reports^9,11^ (Supplementary material). ACQUITY UPLC system (Waters, UK) having ACQUITY UPLC BEH C18 column (Waters, UK)(150mm X 2.1mm X 1.7µm) was used to perform liquid chromatography. A gradient of two mobile phases A (0.1% Formic Acid in WATER), and B (0.1% Formic Acid in Acetonitrile) were used for the chromatographic separation. Further, mass spectrometric detection was performed by using SYNAPT G2 QTOF (Waters, UK) having an electrospray ionization (ESI) source.

### Database searching and analysis

The observed proteins were matched to *Pseudomonas* protein database which was downloaded from Swiss-Prot through PLGS software 3.0.2. The data analysis was performed in triplicates using the following parameters: peptide tolerance = 50 ppm, fragment tolerance = 100 ppm, minimum no. of fragment matches (for Proteins) = 5, minimum no. of fragment matches (for peptides) = 2,, minimum no. of peptide matches (for proteins) = 2, No. of missed cleavages = 2 and modifications include oxidation-m and carbamidomethyl-c. The FDR (false discovery rate) was set as <1% on both protein and peptide levels. The data were normalized across the conditions including the replicates using spectral counts method^40,41^. For identifying differentially expressed proteins, the normalized peptide matches of the treatment were divided by that of control conditions. Statistical analysis was performed through pair-wise Student’s t-test (p ≤ 0.05).

### Construction of the PPI network

Among the total expressed proteins, only two types of proteins were selected for the construction of the PPI (protein-protein interactions) Networks: (1) unique and (2) which showed 2-fold or higher change in their expression. PPI information of proteins was obtained from the String database (version 9.1; http://string-db.org/) having the confidence score > 0.7. It was further imported in Cytoscape (version 3.6.0; http://www.cytoscape.org/) and the union calculation was performed, followed by removal of the duplicated edges by using Advanced Network Merge^40,42^.

### Gene ontology (GO) enrichment analysis

The ontologies and their genes from the PPI network of selected proteins were identified using BiNGO plugin (version 3.0.3; http://apps.cytoscape.org/apps/bingo) for the Cytoscape which are: molecular functions, biological processes, and cellular components. For a detailed description of the biological process, the ClueGo plugin (version 2.5.0; http://apps.cytoscape.org/apps/cluego) of Cytoscape was used to integrate several ontology sources by extracting the non-redundant biological information from several databases *viz*. KEGG, REACTOME, GO, Wiki Pathways and BioCarta^40,43^.

### Statistical analysis

Significance was determined using unpaired two-tailed t-test or linear regression analysis (GraphPad Prism software, version 6.01). Differences were noted as significant *p ≤ 0.05 for t-test or linear regression analysis. The scatter plot analysis was performed by using the software PERMANOVA^44^_._

## Supplementary information

Supplementary Material

## Data Availability

The datasets used and/or analyzed during the current study are available from the corresponding authors on reasonable request.
