# Supplementary material for: Differential protein profiling of soil diazotroph Rhodococcus qingshengii S10107 towards low-temperature and nitrogen deficiency
Source: Sci Rep. 2019 Dec 30;9:20378. doi: 10.1038/s41598-019-56592-8 (PMC6937269; doi:10.1038/s41598-019-56592-8)
Supplement: Supplementary file 1 — Supplementary Material [file 41598_2019_56592_MOESM1_ESM.docx]

**Differential protein profiling of soil diazotroph *Rhodococcus qingshengii* S10107 towards low-temperature and nitrogen deficiency**

Deep Chandra Suyal^a,b^, Divya Joshi^b^, Saurabh Kumar^b^, Ravindra Soni^c^, Reeta Goel^b^*

^a^Department of Microbiology, Akal College of Basic Sciences, Eternal University, Baru Sahib

Sirmaur- 173101, Himachal Pradesh, India

^b^Department of Microbiology, College of Basic Sciences and Humanities, G.B.Pant University of Agriculture and Technology, Pantnagar-263145, Uttarakhand, India

^c^Department of Agricultural Microbiology, College of Agriculture, Indira Gandhi Krishi Viswavidyalaya, Raipur, C.G., India

**^*^ Corresponding author**

E-mail: [rg55@rediffmail.com](mailto:rg55@rediffmail.com)

Ph: +91-9837252162

**(Supplementary Material)**

**Section SM1.**

**Protein Extraction:**

Single colony of both the strains strain were inoculated in 500 ml of broth, separately and grown overnight in “Nutrient broth and Burk’s medium” representing two different conditions - low temperature nitrogen sufficient condition (NB) and low temperature nitrogen fixing condition (BM), respectively. Cultures were harvested at 10,000 rpm for 10 min and pellets were washed twice with Normal saline solution. Pellets were then dissolved in 4ml of 0.1M Phosphate buffer saline (chilled) and sonicated for 3 min in ice. One mM PMSF (100μl) was added to the sonicated pellets. Cell suspension was centrifuged for 45 min at 10,000 rpm. The supernatants were lyophilized and were send to Sandor Proteomics Pvt Ltd, Hyderabad for further analysis.

**
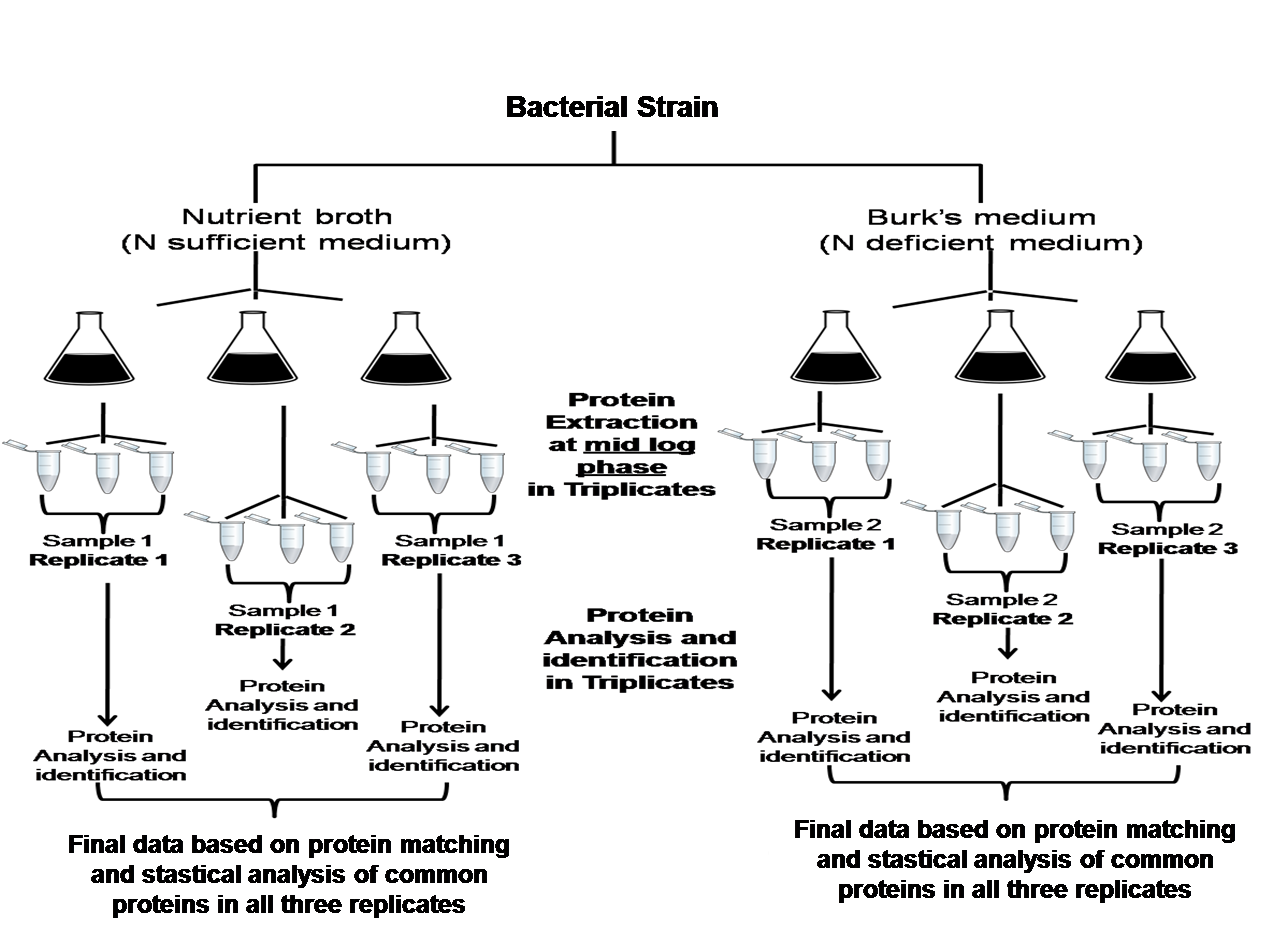
**

**Fig SM1. Methodology used in the study**

**BM1 BM2 BM3**

**(A)**

**NB1 NB2 NB3**

**(B)**

**Fig SM2.** LCMS Chromatogram of *Rhodococcus quingshenghii* S10107strain grown under Nitrogen deficient (A) and Nitrogen sufficient (B) conditions. Analysis was performed in triplicates. Cells were harvested at mid-logarithmic phase.

**Table SM1** Functional characterization of up-regulated and down-regulated selected proteins during low-temperature N deficient condition by *R. qingshengii* S10107 as revealed by LC-MS/MS analysis. The data were processed in triplicate.

| **S.**  **No.** | **Genes** | **Proteins** | **Uniprot ID** | **Biological Functions** | **Fold Change* (BM/NB)** |
| --- | --- | --- | --- | --- | --- |
| **Upregulated proteins (BM)** | | | | | |
|  | *uvr*A1  (ABG92170.1) | UvrABC system protein A | Q0SI39 | DNA repair system | 2.40 ± 0.11 |
|  | *bphy*_3511 ^MP^  (ABG92352.1) | Acetaldehyde dehydrogenase 1 | B2JLM7 | Aromatic hydrocarbons catabolism | 2.89 ± 0.01 |
|  | *leu*D1 ^AAB/MP/SM^ | 3-isopropylmalate dehydratase | Q98E51 | Leucine biosynthesis | 3.47 ± 0.05 |
|  | *upp*P ^MP/NM^ | Undecaprenyl-diphosphatase | Q89WH1 | Peptidoglycan synthesis | 3.51 ± 0.11 |
|  | *xer*D  (ABG92760.1) | Tyrosine recombinase XerD | Q98FX8 | Cell division | 3.86 ± 0.20 |
|  | *pyr*G ^MP/NM^ | CTP synthase | B7KF08 | Glutamine metabolic process | 4.11 ± 0.12 |
|  | *arg*F ^AAB/MP/NM/SM^ | Ornithine carbamoyltransferase | Q8YMM6 | Arginine biosynthetic process | 4.33 ± 0.01 |
|  | *arg*C ^AAB/SM^ | N-acetyl-gamma-glutamyl-phosphate reductase | B7JY20 | Arginine biosynthetic process | 4.39 ± 0.03 |
|  | *phe*T | Phenylalanine--tRNA ligase beta subunit | Q8YMH5 | Phenylalanyl-tRNA aminoacylation | 4.39 ± 0.11 |
|  | *phe*S | Phenylalanine--tRNA ligase alpha subunit | A0A1D8TAJ1 | Phenylalanyl-tRNA aminoacylation | 4.63 ± 0.21 |
|  | *coa*E ^MP^ | Dephospho-CoA kinase | Q98DY2 | Coenzyme A biosynthetic process | 4.71 ± 0.24 |
|  | *lgt* ^MP/NM^ | Prolipoprotein diacylglyceryl transferase | A9HBY2 | Lipoprotein biosynthetic process | 4.97 ± 0.03 |
|  | *his*F ^AAB/ MP/NM^ | Imidazole glycerol phosphate synthase subunit | A8HYT7 | Histidine biosynthetic process | 5.00 ± 0.01 |
|  | *bio*D ^MP/NM^ | ATP-dependent dethiobiotin synthetase | B7K5E6 | Biotin biosynthesis | 5.22 ± 0.07 |
|  | *mur*C ^MP/NM^ | UDP-N-acetylmuramate--L-alanine ligase | B2JHF9 | Cell division | 5.36 ± 0.01 |
|  | *mur*E1 | UDP-N-acetylmuramoyl-L-alanyl-D-glutamate--2,6-diaminopimelate ligase | Q89FU2 | Cell division | 5.41 ± 0.02 |
|  | *mloA* ^MP/NM/SM^ | Protein MloA | Q8RN11 | ATP-binding | 5.48 ± 0.05 |
|  | *lip*B ^MP/NM^ | Octanoyltransferase | Q89JM6 | Protein lipoylation | 5.54 ± 0.13 |
|  | *rec*O | DNA repair protein RecO | Q985A3 | DNA repair | 5.83 ± 0.04 |
|  | *ybe*Y ^MP/NM^ | Endoribonuclease YbeY | Q98BK1 | Ribosome biogenesis | 5.91 ± 0.03 |
|  | *sat*  (ABG93077.1) | Sulfate adenylyltransferase | B7JVS6 | Sulfate assimilation | 5.97 ± 0.02 |
|  | AZC2303 ^AAB/MP/NM^  (ABG93111.1) | Putative glutamate--cysteine ligase | A8I5N7 | Amino acid biosynthetic process | 6.02 ± 0.02 |
|  | *pro*B ^AAB/MP/NM^ | Glutamate 5-kinase | B2JHD6 | L-proline biosynthetic process | 6.13 ± 0.11 |
|  | *clp*X | ATP-dependent Clp protease ATP-binding ClpX | N1M9I2 | Protein folding | 6.13± 0.03 |
|  | *mlr*4626  (ABG93250.1) | dITP/XTP pyrophosphatase | Q98DN4 | Nucleotide metabolic process | 6.18 ± 0.05 |
|  | *pnc*B  (ABG93260.1) | Nicotinate phosphoribosyltransferase | Q89SS3 | NAD biosynthetic process | 6.19 ± 0.02 |
|  | *atp*A ^MP/NM^ | ATP synthase subunit alpha | Q98EV6 | ATP synthesis | 6.21 ± 0.02 |
|  | *atp*H ^MP/NM^ | ATP synthase subunit delta | A8HS18 | ATP synthesis | 6.28 ± 0.03 |
|  | *thr*B ^AAB/MP/SM^ | Homoserine kinase | A9HS91 | Amino-acid biosynthesis | 6.29 ± 0.01 |
|  | *tuf*A ^MP/NM^ | Elongation factor Tu | C0ZVT7 | Protein biosynthesis | 6.34 ± 0.02 |
|  | *rpl*J ^MP/NM^ | 50S ribosomal protein L10 | A9H3S7 | Ribosome biogenesis | 6.37 ± 0.04 |
|  | *hem*L ^MP/NM/SM^ | Glutamate-1-semialdehyde 2,1-aminomutase | B8HYK1 | Porphyrin biosynthesis | 6.39 ± 0.02 |
|  | *Leu*D ^MP/SM^  (ABG93857.1) | 3-isopropylmalate dehydratase small subunit | Q98E51 | Amino-acid biosynthesis | 6.40 ± 0.06 |
|  | *gpm*A ^MP/NM/SM^ | 2,3-bisphosphoglycerate-dependent phosphoglycerate mutase | B2JC95 | Gluconeogenesis | 6.48 ± 0.05 |
|  | *ask*A ^MP^ | Acetate kinase | B8HVB6 | Acetyl-coa biosynthetic process | 6.49 ± 0.09 |
|  | *th*yA ^MP/NM^ | Thymidylate synthase | Q0SEI1 | Nucleotide biosynthesis | 6.54 ± 0.11 |
|  | *dha*A  (ABG94684.1) | Haloalkane dehalogenase | P59337 | Haloalkane dehalogenase activity | 6.58 ± 0.21 |
|  | *fbp*C1 | Fe(3+) ions import ATP-binding protein FbpC | Q98G43 | Ion transport, Iron transport, | 6.59 ± 0.06 |
|  | *rps*F ^MP/NM^ | 30S ribosomal protein S6 | B7JXP0 | Ribosomal protein | 6.60 ± 0.11 |
|  | *tkt* ^MP^  (ABG95643.1) | Transketolase | Q8YRU9 | Transketolase activity | 6.60 ± 0.02 |
|  | *rbs*A | Ribose import ATP-binding protein RbsA | Q0S9A4 | Sugar transport | 6.63 ± 0.04 |
|  | *rsg*A  (ABG95959.1) | Small ribosomal subunit biogenesis GTPase RsgA | B5Y341 | Ribosomal small subunit biogenesis | 6.67 ± 0.01 |
|  | Rwratislav_15378 | Protein-L-isoaspartate O-methyltransferase | L2TM59 | Methyltransferase activity | 6.67 ± 0.02 |
|  | *tgt* | Queuine tRNA-ribosyltransferase | A1K3W9 | Queuosine biosynthesis, tRNA processing | 6.69 ± 0.12 |
|  | *acc*C ^MP^  (ABG96013.1) | Biotin carboxylase | Q06862 | malonyl-CoA biosynthetic process | 6.72 ± 0.02 |
|  | *til*S ^MP/NM^ | tRNA(Ile)-lysidine synthase | A1K3X8 | tRNA modification | 6.78 ± 0.11 |
|  | *pan*D ^MP/NM/SM^ | Aspartate 1-decarboxylase | Q8YR79 | Alanine biosynthetic process | 6.81 ± 0.13 |
|  | *eca*A (ABG96235.1) | Carbonic anhydrase | P94170 | Carbonate dehydratase activity | 6.86 ± 0.01 |
|  | *clp*P (ABG96410.1) | ATP-dependent Clp protease proteolytic subunit | A8HYF2 | Degradation of misfolded proteins | 6.87 ± 0.14 |
|  | *hut*I ^MP^ | Imidazolonepropionase | B2JCJ1 | Amino-acid degradation | 6.93 ± 0.07 |
|  | *pur*D ^MP/SM^ | Phosphoribosylamine--glycine ligase | C1BBR1 | Purine metabolism | 6.98 ± 0.02 |
|  | *dct*A2 (ABG96587.1) | C4-dicarboxylate transport protein | Q986R8 | Dicarboxylic acid transport | 6.98 ± 0.11 |
|  | *trm*B ^MP/NM^ | tRNA (guanine-N(7)-)-methyltransferase | Q89WA4 | tRNA modification | 7.02 ± 0.03 |
|  | *pck*G ^MP/SM^ | Phosphoenolpyruvate carboxykinase | B2JJT8 | Gluconeogenesis | 7.08 ± 0.01 |
|  | *lld*D ^MP^ (ABG96995.1) | L-lactate dehydrogenase | A8HTC9 | Lactate oxidation | 7.29 ± 0.03 |
|  | *pyr*E ^MP^ | Orotate phosphoribosyltransferase | P0A7E3 | Pyrimidine metabolism | 7.33 ± 0.02 |
|  | *suc*C ^MP/SM^ | Succinate--CoA ligase | A1KAU3 | Tricarboxylic acid cycle | 7.63 ± 0.03 |
|  | *ure*A ^MP/NM^ | Urease subunit gamma | Q8YQZ3 | Urea degradation and Nitrogen metabolism | 7.64 ± 0.07 |
|  | *ure*F ^MP^ | Urease accessory protein UreF | B5XU25 | Nitrogen compound metabolic process | 7.78 ± 0.11 |
|  | *ure*G ^MP/NM^ | Urease accessory protein UreG | A1KBB1 | Nitrogen compound metabolic process | 7.83 ± 0.05 |
|  | *rpl*Y ^MP^ | 50S ribosomal protein L25 | B7KCY0 | Translation | 8.01 ± 0.02 |
|  | *eno* ^MP/NM/SM^ | Enolase | B2JIX0 | Glycolysis | 8.14 ± 0.02 |
|  | *xse*B | Exodeoxyribonuclease 7 small subunit | Q89RW0 | DNA catabolic process | 8.26 ± 0.06 |
|  | *htp*G | Chaperone protein HtpG | Q0S467 | Protein folding | 8.36 ± 0.04 |
|  | *nuo*C ^MP^ | NADH-quinone oxidoreductase subunit C | Q98KQ7 | Transport | 8.39 ± 0.03 |
|  | *nuo*D ^MP^ | NADH-quinone oxidoreductase subunit D | A9HRT9 | Transport | 8.42 ± 0.02 |
|  | *dap*D ^AAB^ | 2,3,4,5-tetrahydropyridine-2,6-dicarboxylate N-succinyltransferase | B2JID7 | Lysine biosynthetic process | 8.45 ± 0.03 |
|  | *dea*D | ATP-dependent RNA helicase DeaD | V9XLR7 | ribosome biogenesis, translation initiation etc. | 8.59 ± 0.02 |
|  | *mur*I (ABG97828.1) | Glutamate racemase | Q98NP9 | Regulation of cell shape | 8.63 ± 0.07 |
|  | *rps*C ^MP/NM^ | 30S ribosomal protein S3 | A0A0C2ZWD4 | Translation | 8.78 ± 0.01 |
|  | *rpl*N ^MP/NM^ | 50S ribosomal protein L14 | Q0S3G6 | Translation | 8.83 ± 0.03 |
|  | *rpl*E | 50S ribosomal protein L5 | I0W8V4 | Translation | 8.89 ± 0.06 |
|  | *rpl*O1 ^MP/NM^ | 50S ribosomal protein L15 | Q89JA3 | Translation | 8.93 ± 0.02 |
|  | *rpl*M ^MP/NM^ | 50S ribosomal protein L13 | Q982W8 | 50S ribosomal assembly | 8.99 ± 0.01 |
|  | *Glm*M ^MP^ | Phosphoglucosamine mutase | Q98F91 | Carbohydrate metabolic process | 9.13 ± 0.10 |
|  | *glm*S ^MP^ | Glutamine--fructose-6-phosphate aminotransferase | P59362 | Glutamine metabolic process | 9.16 ± 0.17 |
|  | *alr* ^AAB^ | Alanine racemase | W8HAH8 | D-alanine biosynthetic process | 9.21 ± 0.11 |
|  | *tsa*D ^MP/NM^ | tRNA N6-adenosine threonylcarbamoyltransferase | Q8Z0I6 | tRNA processing | UBM** |
|  | *dna*E | Error-prone DNA polymerase | Q98E34 | DNA repair | UBM** |
|  | *pcy*A (ABG98238.1) | Phycocyanobilin:ferredoxin oxidoreductase | Q93TN0 | Phytochromobilin biosynthetic process | UBM** |
|  | *ddl*A ^MP/NM^ | D-alanine--D-alanine ligase | B2JHF8 | Peptidoglycan biosynthesis | UBM** |
|  | *rplS* ^MP/NM^ | 50S ribosomal protein L19 | B7JWU1 | Translation | UBM** |
|  | *rnh*B ^MP/NM^ | Ribonuclease HII | A0A1H7FMX8 | RNA catabolic process | UBM** |
|  | *dxr* ^MP/SM^ | 1-deoxy-D-xylulose 5-phosphate reductoisomerase | B7K5G6 | Isoprenoid biosynthesis | UBM** |
|  | *mqo* ^MP/SM^ | Probable malate:quinone oxidoreductase | Q89XM4 | Tricarboxylic acid cycle | UBM** |
|  | *rim*P | Ribosome maturation factor RimP | T5I446 | Maturation of 30S ribosomal subunits | UBM** |
|  | *hem*E ^MP/NM/SM^ | Uroporphyrinogen decarboxylase | Q0S1F6 | Porphyrin biosynthesis | UBM** |
|  | *aro*B ^AAB/MP/SM^ | 3-dehydroquinate synthase | Q0S0N0 | Aromatic amino acid biosynthesis | UBM** |
|  | *nusB*  ^MP/NM^ | Transcription antitermination protein NusB | B7K4P4 | Transcription of ribosomal RNA | UBM** |
|  | *pyr*R ^MP^ | Bifunctional protein PyrR | A0A0E3VC96 | Nucleoside metabolic process | UBM** |
|  | *pyr*C ^MP^ | Dihydroorotase | A1K3T5 | Pyrimidine bisynthesis | UBM** |
|  | *tpi*A ^MP/NM/SM^ | Triosephosphate isomerase | Q8YP17 | Gluconeogenesis | UBM** |
|  | *tal* ^MP/SM^ | Transaldolase | P58561 | Pentose-phosphate pathway | UBM** |
|  | *nif*H | Nitrogenase iron protein | C7SI80 | Nitrogen fixation | UBM** |
|  | *nif*A | *nif*-specific regulatory protein | P56266 | Activation of most *nif* operons | UBM** |
|  | *nif*L | Nitrogen fixation regulatory protein | P06772 | Regulation of nitrogen fixation | UBM** |
|  | *nif*B | FeMo cofactor biosynthesis protein | P09825 | Biosynthesis of the iron-molybdenum cofactor | UBM** |
|  | *nif*D | Nitrogenase molybdenum-iron protein | P06120 | Nitrogen fixation | UBM** |
|  | *nif*K | Nitrogenase molybdenum-iron protein | P07329 | Nitrogen fixation | UBM** |
|  | *nir*S | Nitrite reductase | P24474 | Nitrite reduction | UBM** |
|  | *cow*N | N(2)-fixation sustaining protein CowN | C1DIY8 | Protecting nitrogenase from CO | UBM** |
|  | *coo*A | Carbon monoxide oxidation transcription regulator | C1DIY7 | CO regulator | UBM** |
| **Downregulated proteins (NB)** | | | | | |
|  | *lex*A  (ABG92407.1) | LexA repressor | U0E9W7 | SOS response | 0.44 ± 0.01 |
|  | *dad*A1 | D-amino acid dehydrogenase 1 | Q9HTQ0 | D-alanine catabolic process | 0.43 ± 0.02 |
|  | *dap*A1 ^AAB/MP/SM^ | 4-hydroxy-tetrahydrodipicolinate synthase | A0A1H4N6J7 | Amino-acid biosynthesis | 0.43 ± 0.01 |
|  | *arc* ^NM^ | Proteasome-associated ATPase | Q0SIF4 | Chaperone | 0.42 ± 0.02 |
|  | *glc*B ^MP^ | Acyl-[acyl-carrier-protein]--UDP-N-acetylglucosamine O-acyltransferase | A8I491 | Lipid A biosynthesis | 0.42 ± 0.03 |
|  | *nad*K ^MP/NM^ | NAD kinase | Q0SI70 | NADP biosynthetic process | 0.41 ± 0.03 |
|  | *tyr*S ^AAB^ | Tyrosine--tRNA ligase | Q98NS5 | Protein biosynthesis | 0.41 ± 0.01 |
|  | *arg*J ^AAB/MP/SM^ | Arginine biosynthesis bifunctional protein ArgJ 2 | Q8YPF9 | Arginine biosynthesis | 0.41 ± 0.02 |
|  | *rpl*T ^NM^ | 50S ribosomal protein L20 | Q0SI47 | translation | 0.40 ± 0.01 |
|  | *nad*A ^MP^ | Quinolinate synthase A | P11458 | NAD(+) biosynthesis | 0.40 ± 0.01 |
|  | *rha*1_ro01056 ^MP/SM^ (ABG92883.1) | Probable glycogen debranching enzyme | Q0SHV3 | glycogen catabolic process | 0.39 ± 0.02 |
|  | *ile*S ^AAB^ | Isoleucine--tRNA ligase | U0FHY2 | Protein biosynthesis | 0.39 ± 0.03 |
|  | *mur*E1 | UDP-N-acetylmuramoyl-L-alanyl-D-glutamate--2,6-diaminopimelate ligase | Q0SHR6 | regulation of cell shape | 0.38 ± 0.01 |
|  | *rha*1_ro01133 ^MP^ (ABG92960.1) | Ubiquinol-cytochrome c reductase cytochrome b subunit | Q0SHM6 | electron transport chain | 0.38 ± 0.03 |
|  | *pan*B ^MP/NM/SM^ | 3-methyl-2-oxobutanoate hydroxymethyltransferase | Q0SHJ0 | Pantothenate biosynthesis | 0.37 ± 0.01 |
|  | *rha*1_ro01186 (ABG93013.1) | Protein-tyrosine-phosphatase | Q0SHH3 | protein tyrosine phosphatase activity | 0.37 ± 0.03 |
|  | *era* | GTPase Era | Q0SHC4 | ribosomal small subunit biogenesis | 0.37 ± 0.02 |
|  | *pro*A ^AAB/MP/NM/SM^ | Gamma-glutamyl phosphate reductase | Q89X85 | Proline biosynthesis | 0.37 ± 0.01 |
|  | *aro*E1 ^AAB/MP/SM^ | Shikimate dehydrogenase | Q98DY3 | Aromatic amino acid biosynthesis | 0.36 ± 0.01 |
|  | *acp*S ^MP^ (ABG93244.1) | Holo-[acyl-carrier-protein] synthase | Q0SGU2 | fatty acid biosynthetic process | 0.36 ± 0.01 |
|  | *rha*1_ro01444 (ABG93262.1) | Probable ATP-dependent helicase | Q0SGS4 | nucleic acid binding | 0.36 ± 0.02 |
|  | *rha*1_ro01447 ^MP/SM^ (ABG93265.1) | Glycogen phosphorylase | Q0SGS1 | carbohydrate metabolic process | 0.36 ± 0.03 |
|  | *prm*C | Release factor glutamine methyltransferase | Q98G94 | peptidyl-glutamine methylation | 0.35 ± 0.01 |
|  | *rha*1_ro01670  (ABG93483.1) | Probable restriction modification enzyme subunit | Q0SG53 | restriction modification enzyme | 0.35 ± 0.02 |
|  | nha*A1* | Na(+)/H(+) antiporter NhaA 1 | Q0SG15 | cell redox homeostasis | 0.35 ± 0.01 |
|  | *met*N1 | Methionine import ATP-binding protein MetN 1 | Q0SFY5 | methionine transport | 0.35 ± 0.03 |
|  | *fus*A ^NM^  (ABG93733.1) | Elongation factor G | Q0SFF3 | Protein biosynthesis | 0.35 ± 0.02 |
|  | *hbd*A ^MP^ | 3-hydroxybutyryl-CoA dehydrogenase | Q45223 | Lipid metabolism | 0.34 ± 0.02 |
|  | *spe*E ^MP^ | Polyamine aminopropyltransferase | Q0SEB7 | Polyamine biosynthesis | 0.34 ± 0.03 |
|  | *rpi*A ^MP/SM^ | Ribose-5-phosphate isomerase A | B7K6D3 | pentose phosphate pathway | 0.32 ± 0.01 |
|  | *rha*1_ro04101 ^MP^  (ABG95898.1) | Probable acyl-[acyl-carrier-protein]--UDP-N-acetylglucosamine O-acyltransferase | Q0S988 | lipid biosynthetic process | 0.32 ± 0.03 |
|  | *rec*R | Recombination protein RecR | Q0S8Y4 | DNA repair | 0.32 ± 0.01 |
|  | *rha*1_ro04386 ^MP^  (ABG96172.1) | Possible aliphatic amidase | Q0S8G4 | [nitrogen compound metabolic process](https://www.ebi.ac.uk/QuickGO/term/GO:0006807) | 0.32 ± 0.01 |
|  | *ppa* | Inorganic pyrophosphatase | Q0S8F0 | inorganic diphosphatase activity | 0.31 ± 0.01 |
|  | *fts*H | ATP-dependent zinc metalloprotease FtsH | A0A059MKS5 | cell division | 0.31 ± 0.01 |
|  | *hut*U ^MP^ | Urocanate hydratase | Q89GV4 | Histidine metabolism | 0.31 ± 0.02 |
|  | *pur*M ^MP/SM^ | Phosphoribosylformylglycinamidine cyclo-ligase | Q0S760 | Purine biosynthesis | 0.29 ± 0.03 |
|  | *pst*S | Phosphate-binding protein PstS | Q98FL2 | Phosphate transport | 0.28 ± 0.01 |
|  | *rha*1_ro05098 ^MP/SM^ (ABG96879.1) | 3-demethylubiquinone-9 3-O-methyltransferase | Q0S6F7 | ubiquinone biosynthetic process | 0.28 ± 0.02 |
|  | *fab*H | 3-oxoacyl-[acyl-carrier-protein] synthase 3 | Q982Z8 | fatty acid biosynthesis | 0.27 ± 0.03 |
|  | *met*S1 | Methionine--tRNA ligase | Q0S4U4 | methionyl-tRNA aminoacylation | 0.27 ± 0.01 |
|  | *Ksg*A ^NM^ | rRNA small subunit methyltransferase A | A0A379MN38 | rRNA processing | 0.27 ± 0.03 |
|  | *ure*B ^MP^ | Urease subunit beta | Q98CY6 | urease activity | 0.27 ± 0.02 |
|  | *glm*U ^MP/NM^ | Bifunctional protein GlmU | Q0S4N3 | lipid A biosynthetic process | 0.26 ± 0.03 |
|  | *rha*1_ro05847 (ABG97624.1) | FAD:protein FMN transferase | Q0S4B2 | protein flavinylation | 0.26 ± 0.01 |
|  | *rpl*C ^NM^ | 50S ribosomal protein L3 | X0QHH3 | translation | 0.26 ± 0.02 |
|  | *rpo*A ^MP/NM^ | DNA-directed RNA polymerase subunit alpha | Q0S3E7 | transcription | 0.26 ± 0.01 |
|  | *rha*1_ro06238 ^MP/SM^ (ABG98015.1) | Isocitrate dehydrogenase [NADP] | Q0S371 | tricarboxylic acid cycle | UNB*** |
|  | *glp*K ^MP/SM^ | Glycerol kinase | A1K962 | glycerol metabolic process | UNB*** |
|  | *aro*A ^AAB/MP/NM/SM^ | 3-phosphoshikimate 1-carboxyvinyltransferase | C1B1F1 | Aromatic amino acid biosynthesis | UNB*** |
|  | *moa*A ^MP/NM^ | GTP 3',8-cyclase | A0A143Q879 | Molybdenum cofactor biosynthesis | UNB*** |
|  | *glt*X2 ^MP/NM/SM^ | Glutamate--tRNA ligase | A0A1F2PR92 | Protein biosynthesis | UNB*** |
|  | *coa*D ^MP/NM^ | Phosphopantetheine adenylyltransferase | B5XTG9 | coenzyme A biosynthetic process | UNB*** |
|  | *rnc* | Ribonuclease 3 | Q0S2E1 | mRNA processing | UNB*** |
|  | *cob*B ^MP^ | Hydrogenobyrinate a,c-diamide synthase | Q98KP1 | Cobalamin biosynthesis | UNB*** |
|  | *mia*A ^NM/SM^ | tRNA dimethylallyltransferase | B2JGD1 | tRNA processing | UNB*** |
|  | *thr*S ^AAB/NM^ | Threonine--tRNA ligase | Q0S1E2 | Protein biosynthesis | UNB*** |
|  | *ruv*A | ATP-dependent DNA helicase RuvA | B7JZG7 | DNA repair | UNB*** |
|  | *apt* ^MP/NM^ | Adenine phosphoribosyltransferase | Q0S1C1 | Purine salvage | UNB*** |
|  | *his*S ^AAB/NM^ | Histidine--tRNA ligase | Q0S1B6 | Protein biosynthesis | UNB*** |
|  | *asp*S ^AAB/NM^ | Aspartate--tRNA(Asp/Asn) ligase | Q0S0P3 | Protein biosynthesis | UNB*** |
|  | *aro*K ^AAB/MP/NM/SM^ | Shikimate kinase | Q98FY0 | Aromatic amino acid biosynthesis | UNB*** |
|  | *ku* | Non-homologous end joining protein Ku | A9H271 | DNA repair | UNB*** |
|  | *acn*A2 ^MP/SM^ | Aconitate hydratase | Q0S0G5 | aconitate hydratase activity | UNB*** |
|  | *hem*Z ^MP/ SM^ | Ferrochelatase | Q0S0F7 | heme biosynthetic process | UNB*** |

(i) *Significantly expressed (P < 0.05); **UBM- Unique to BM; ***UNB- Unique to NB

(ii) Superscript & underlined words represent the functional enrichments of the PPI network (only major categories are shown) i.e. AAB- amino acid biosynthesis; MP- metabolic pathway; NM- nitrogen metabolism; SM- Secondary metabolites

**Table SM 2**. Comparative expressional pattern of the consistently expressed proteins under low temperature N_2_ deficient conditions as revealed by different studies *viz*. *Rhodococcus quingshenghii* S10107 (present study) *Dyadobacter* *psychrophilus* B2 and *Pseudomonas jessenii* MP1 (Suyal et al. 2017); *P. palleroniana* N26-GL (Suyal et al. 2018); *P. palleroniana* N26-GB (Soni et al. 2015) and *P. migulae* S10724 (Suyal et al. 2014). Up-regulated and down-regulated proteins are being represented by 1 and -1, respectively; while, absent proteins are marked as 0.

| **Proteins** | **Genes** | ***R. quingshenghii* S10107** | ***P. migulae* S10724** | ***P. palleroniana* N26-GB** | ***D.* *psychrophilus* B2** | ***P. jessenii* MP1** | ***P. palleroniana* N26-GL** | **pI** | **MW** |
| --- | --- | --- | --- | --- | --- | --- | --- | --- | --- |
| UDP-3-O-acylglucosamine N-acyltransferase | *glc*B | 1.00 | 0.00 | 0.00 | -1.00 | 0.00 | 0.00 | 6.1 | 36.335 |
| N-acetyl-gamma-glutamyl-phosphate reductase | *arg*C | 1.00 | 0.00 | 0.00 | -1.00 | 0.00 | 0.00 | 5.38 | 34.931 |
| Dephospho-CoA kinase | *coa*E | 1.00 | 0.00 | 0.00 | 0.00 | 1.00 | 0.00 | 4.3 | 16.867 |
| Endoribonuclease YbeY | *ybe*Y | 1.00 | 0.00 | 0.00 | -1.00 | 0.00 | 0.00 | 4.18 | 17.145 |
| Glutamate 5-kinase | *pro*B | 1.00 | 0.00 | -1.00 | 0.00 | 0.00 | 0.00 | 5.99 | 30.110 |
| ATP-dependent Clp protease ATP-binding ClpX | *clp*X | 1.00 | 1.00 | 0.00 | -1.00 | 0.00 | 0.00 | 5.44 | 46.230 |
| ATP synthase subunit alpha | *atp*A | 1.00 | 0.00 | 0.00 | 1.00 | 1.00 | 0.00 | 5.3 | 55.450 |
| ATP synthase subunit delta | *atp*H | 1.00 | 0.00 | 1.00 | 0.00 | 0.00 | 0.00 | 5.1 | 19.250 |
| 30S ribosomal protein S6 | *rps*F | 1.00 | 0.00 | 0.00 | 0.00 | 0.00 | 1.00 | 6.24 | 23.568 |
| Protein-L-isoaspartate O-methyltransferase | rwratislav_15378 | 1.00 | 1.00 | 0.00 | 0.00 | 0.00 | 0.00 | 6.08 | 23.699 |
| ATP-dependent Clp protease proteolytic subunit | *clp*P | 1.00 | 0.00 | 1.00 | 0.00 | 0.00 | 0.00 | 4.54 | 22.282 |
| Phosphoenolpyruvate carboxykinase | *pck*G | 1.00 | 0.00 | 0.00 | 0.00 | 0.00 | -1.00 | 6.09 | 68.243 |
| Urease accessory protein UreF | *ure*F | 1.00 | 0.00 | 0.00 | 1.00 | 0.00 | 0.00 | 5.45 | 24.968 |
| Enolase | *eno* | 1.00 | 1.00 | 0.00 | 1.00 | 1.00 | 0.00 | 4.7 | 46.473 |
| Chaperone protein HtpG | *htp*G | 1.00 | 0.00 | 0.00 | 1.00 | 1.00 | 0.00 | 5.33 | 67.209 |
| 50S ribosomal protein L5 | *rpl*E | 1.00 | 0.00 | 0.00 | 0.00 | 0.00 | 1.00 | 9.81 | 21.852 |
| Glutamine--fructose-6-phosphate aminotransferase | *glm*S | 1.00 | 0.00 | 0.00 | 0.00 | 0.00 | 1.00 | 5.69 | 67.534 |
| D-alanine--D-alanine ligase | *ddl*A | 1.00 | 1.00 | 0.00 | 0.00 | 0.00 | 0.00 | 4.46 | 40.789 |
| Ribonuclease HII | *rnh*B | 1.00 | 0.00 | -1.00 | 0.00 | 0.00 | 0.00 | 5.64 | 30.797 |
| Ribosome maturation factor RimP | *rim* P | 1.00 | 0.00 | -1.00 | 0.00 | 0.00 | 0.00 | 4.34 | 17.506 |
| tRNA dimethylallyltransferase | *mia*A | -1.00 | 0.00 | 0.00 | 0.00 | 0.00 | -1.00 | 9.95 | 34.418 |
| Uroporphyrinogen decarboxylase | *hem*E | 1.00 | 0.00 | 0.00 | 0.00 | 0.00 | 1.00 | 5.6 | 40.954 |
| CTP Synthase | *pyr*G | 1.00 | 0.00 | -1.00 | -1.00 | -1.00 | 0.00 | 5.97 | 38.228 |
| Nitrogenase iron protein *nif*H | *nif*H | 1.00 | 0.00 | 0.00 | 0.00 | 0.00 | 1.00 | 4.5264 | 31.949 |
| *nif*-specific regulatory protein *nif*L | *nifL* | 1.00 | 0.00 | 0.00 | 0.00 | 0.00 | 1.00 | 4.78 | 57.827 |
| Nitrogen fixation regulatory protein *nif*A | *nifA* | 1.00 | 0.00 | 0.00 | 0.00 | 0.00 | 1.00 | 8.0244 | 66.751 |
| FeMo cofactor biosynthesis protein *nif*B | *nif*B | 1.00 | 0.00 | 0.00 | 0.00 | 0.00 | 1.00 | 6.3223 | 53.472 |
| Nitrogenase molybdenum-iron protein *nif*D | *nif*D | 1.00 | 0.00 | 0.00 | 0.00 | 0.00 | 1.00 | 5.5649 | 59.087 |
| Nitrogenase molybdenum-iron protein *nif*K | *nif*K | 1.00 | 0.00 | 0.00 | 0.00 | 0.00 | 1.00 | 6.4409 | 58.179 |
| Nitrite reductase | *nir*S | 1.00 | 0.00 | 0.00 | 0.00 | 0.00 | 1.00 | 4.6201 | 65.398 |
| N(2)-fixation sustaining protein CowN | *cow*N | 1.00 | 1.00 | 1.00 | 1.00 | 1.00 | 1.00 | 4.7 | 13.237 |
| Carbon monoxide oxidation transcription regulator | *coo*A | 1.00 | 0.00 | 0.00 | 0.00 | 0.00 | 1.00 | 4.30 | 24.762 |
| ATP-dependent protease | *com* M | 0.00 | 1.00 | 0.00 | 1.00 | 0.00 | 0.00 | 5.15 | 3.110 |
| Isocitrate dehydrogenase kinase/phosphatase | *ace* K | 0.00 | 1.00 | 0.00 | 0.00 | -1.00 | 0.00 | 6.11 | 67.206 |
| *Phosphoglycerate kinase* | *pgk* | 0.00 | 1.00 | 1.00 | 0.00 | 0.00 | 0.00 | 5.62 | 42.250 |
| *GTPase obg* | *obg* | 0.00 | 1.00 | 0.00 | 0.00 | 0.00 | -1.00 | 5.2 | 47.205 |
| Protein GrpE | *grp* E | 0.00 | 1.00 | 0.00 | -1.00 | 0.00 | 0.00 | 6.46 | 21.431 |
| Chaperone protein TorD | *tor* D | 0.00 | 1.00 | 0.00 | 0.00 | 1.00 | 0.00 | 4.63 | 24.813 |
| LexA repressor | *lex* A | -1.00 | 1.00 | 0.00 | 0.00 | 0.00 | 0.00 | 4.98 | 22.678 |
| Adenine phosphoribosyltransferase | *apt* | -1.00 | 1.00 | 0.00 | 0.00 | 0.00 | 0.00 | 5.03 | 19.183 |
| 10 kDa chaperonin | *gro* S | 0.00 | 1.00 | 0.00 | 0.00 | -1.00 | 0.00 | 6.1 | 9.441 |
| Ferredoxin-like protein in nif region | *fdx* N | 0.00 | 1.00 | 0.00 | 0.00 | 1.00 | 0.00 | 4.9 | 6.950 |
| Adapter protein MecA | *mec* A | 0.00 | 1.00 | 0.00 | -1.00 | 0.00 | 0.00 | 4.3 | 26.446 |
| tRNA modification GTPase MnmE | *mnm*E | 0.00 | -1.00 | -1.00 | 0.00 | 0.00 | -1.00 | 4.92 | 51.478 |
| Cell division protein FtsZ | *fts* Z | 0.00 | -1.00 | -1.00 | 0.00 | 0.00 | 0.00 | 4.54 | 38.756 |
| Adenylate kinase | *adk* | 0.00 | -1.00 | 0.00 | 0.00 | -1.00 | 0.00 | 6.74 | 23.705 |
| Cell division protein SepF | *sep* F | 0.00 | -1.00 | 0.00 | 0.00 | -1.00 | 0.00 | 4.32 | 20.913 |
| DNA gyrase inhibitor YacG | *yac* G | 0.00 | 0.00 | 1.00 | 0.00 | 0.00 | 1.00 | 4.18 | 8.160 |
| Ketol-acid reductoisomerase | *ilv*C | 0.00 | 0.00 | 1.00 | -1.00 | 0.00 | -1.00 | 5.77 | 36.122 |
| Ribosome maturation factor RimM | *rim* M | 0.00 | 0.00 | 1.00 | -1.00 | 0.00 | 0.00 | 4.24 | 19.935 |
| Quinolinate synthase A | *nad* A | -1.00 | 0.00 | 1.00 | 0.00 | 0.00 | 0.00 | 4.25 | 41.500 |
| Urocanate hydratase | *hut* U | -1.00 | 0.00 | 1.00 | 0.00 | 0.00 | 0.00 | 5.7 | 60.750 |
| 3-dehydroquinate dehydratase | *aro*Q1 | 0.00 | 0.00 | -1.00 | 0.00 | -1.00 | 0.00 | 6.22 | 16.498 |
| Ribonuclease 3 | *rnc* | -1.00 | 0.00 | -1.00 | 0.00 | 0.00 | 0.00 | 6 | 25.307 |
| Cell division protein ZipA homolog | *zip* A | 0.00 | 0.00 | -1.00 | 0.00 | -1.00 | 0.00 | 5 | 38.094 |
| Argininosuccinate synthase | *arg* G | 0.00 | 0.00 | -1.00 | -1.00 | 0.00 | 0.00 | 4.8 | 44.826 |
| 60 kDa chaperonin groL | *gro* L | 0.00 | 0.00 | 0.00 | 1.00 | 1.00 | 0.00 | 5 | 57.820 |
| Uncharacterized PPE family protein PPE12 | *ppe*12 | 0.00 | 0.00 | 0.00 | 1.00 | 1.00 | 0.00 | 4.59 | 62.693 |
| DNA mismatch repair protein MutL | *mut*L | 0.00 | 0.00 | 0.00 | 1.00 | 1.00 | 1.00 | 6.46 | 67.575 |
| ATP synthase subunit beta | *atp* D | 0.00 | 0.00 | 0.00 | 1.00 | 1.00 | 0.00 | 5.17 | 55.626 |
| Chaperone protein ClpB | *clp*B | 0.00 | 0.00 | 0.00 | 1.00 | 0.00 | -1.00 | 4.95 | 94.438 |
| DNA ligase | *lig* A | 0.00 | 0.00 | 0.00 | 1.00 | -1.00 | 0.00 | 5.01 | 86.516 |
| NAD kinase | *nad* K | -1.00 | 0.00 | 0.00 | -1.00 | 0.00 | 0.00 | 5.91 | 32.276 |
| Leucine--tRNA ligase | *ile*S | -1.00 | 0.00 | 0.00 | -1.00 | 0.00 | -1.00 | 5.42 | 93.198 |
| Translation initiation factor IF-2 | *inf* B | 0.00 | 0.00 | 0.00 | -1.00 | 0.00 | -1.00 | 5.41 | 106.191 |
| Glutamyl-tRNA(Gln) amidotransferase subunit E | *gat* E | 0.00 | 0.00 | 0.00 | -1.00 | -1.00 | 0.00 | 6.6 | 67.590 |
| 3-oxoacyl-[acyl-carrier-protein] synthase 3 | *fab* H | -1.00 | 0.00 | 0.00 | -1.00 | 0.00 | 0.00 | 5.52 | 34.823 |
| Chaperone protein HscA homolog | *hsc* A | 0.00 | 0.00 | 0.00 | 0.00 | 1.00 | -1.00 | 5.12 | 65.688 |
| Arginine biosynthesis bifunctional protein ArgJ | *arg* J | -1.00 | 0.00 | 0.00 | 0.00 | -1.00 | 0.00 | 6.55 | 23.013 |
| Serine--tRNA ligase | *ser*S | 0.00 | 0.00 | 0.00 | 0.00 | -1.00 | -1.00 | 5.41 | 48.009 |
| Glutamate--tRNA ligase | gltX | -1.00 | 0.00 | 0.00 | 0.00 | -1.00 | 0.00 | 5.64 | 52.193 |
| Ribose-5-phosphate isomerase A | *rpi* A | -1.00 | 0.00 | 0.00 | 0.00 | -1.00 | 0.00 | 4.63 | 23.504 |
| Proline--tRNA ligase | *pro*S | 0.00 | 0.00 | 0.00 | 0.00 | -1.00 | 1.00 | 6.41 | 62.551 |
| Histidine--tRNA ligase | *his*S | -1.00 | 0.00 | 0.00 | 0.00 | 0.00 | 1.00 | 4.9116 | 49.899 |
| 50S ribosomal protein L3 | *rpl*C | -1.00 | 0.00 | 0.00 | 0.00 | 0.00 | -1.00 | 10.0796 | 29.033 |
| GTP 3',8-cyclase | *moa* A | -1.00 | 0.00 | 0.00 | 0.00 | 0.00 | -1.00 | 6.2607 | 36.854 |
